# An Independent Study to Compare Compliance, Attitudes, Knowledge, and Sources of Knowledge about Pneumococcal Vaccinations among an Italian Sample of Older Adults

**DOI:** 10.3390/vaccines10040490

**Published:** 2022-03-23

**Authors:** Caterina Suitner, Bruno Gabriel Salvador Casara, Stefania Maggi, Vincenzo Baldo

**Affiliations:** 1 Department of Developmental Psychology and Socialisation, University of Padova, 35121 Padova, Italy; caterina.suitner@unipd.it; 2 Department of Biomedical Science, University of Padova, 35121 Padova, Italy; stefania.maggi@in.cnr.it; 3 Department of Cardiac, Thoracic, Vascular Sciences and Public Health, University of Padova, 35121 Padova, Italy; vincenzo.baldo@unipd.it

**Keywords:** *Streptococcus pneumoniae*, vaccine, vaccine literacy, vaccine hesitancy, vaccine coverage, risk perception, information sources, optimistic bias, elderly

## Abstract

Background: *Streptococcus pneumoniae* is one of the leading causes of pneumoniae deaths, especially among elderly individuals, with the vaccine being the primary prevention instrument. However, information on national vaccine coverage among the elderly population is scarce and sparse. Methods: A survey involved a representative sample of Italians older than 65 years (*n* = 600), who agreed to participate in the study through a phone interview. Participants’ self-reported pneumococcal vaccination status, vaccine literacy, information source, and risk perception related to the infection and to vaccines-adverse reactions were assessed. Results: The reported vaccination status is very low (11.2%), with respondents largely uninformed about vaccination opportunities. The results also show that the predominant (and most effective) source of information is healthcare providers, with vaccine hesitancy being positively linked to risk perception related to disease and negatively linked to risk perception of vaccine adverse reactions. Conclusions: This study suggests the need to collect data to systematically monitor vaccination coverage and calls for information campaigns to improve elderly literacy to increase vaccination uptake.

## 1. Introduction

*Streptococcus pneumoniae* (from now on mentioned as Pneumococcus) is a widespread bacterium, responsible for serious infections, especially among adults over 65 years. For example, in Italy, during 2020, the incidence rate of pneumococcal infection was 2.04 on 100.000 for people older than 65, where the incidence rate was 0.84 for the entire population (ISS, 2020). In the WHO 2018 report, Pneumococcus is indicated as the most frequent cause of severe pneumonia and pneumonia deaths worldwide, with the vaccine being the primary prevention instrument to achieve healthy aging [1].

Currently, there are two available vaccines for the prevention of pneumococcal disease in adults, namely, the 23-valent pneumococcal polysaccharide vaccine (PPSV-23) and the 13-valent pneumococcal conjugate vaccine (PCV-13). Recommendations vary widely across the world, even within Europe [2], with important consequences on vaccine coverage and ultimately on the health of elderly persons [3]. Many countries (including Italy) recommend vaccination to people older than 65 years, with Healthy People (2020) recommending coverage of 90%. Yet very little is known about the actual coverage in many countries. Only a few studies have addressed this issue, reporting high variability across nations. For example, in Sweden, the reported coverage was 50% in 1998–2000, in the USA it was 59% in 1997 and 71% in 1999, in Israel it was 20% in 2000/2001 and 27.9% in 2001/2002 [4], in Spain it was 52.8% in 2017 [5]. To the best of our knowledge, the only available information about the coverage in Italy is from a web-based survey where a national convenience sample of volunteers was considered, where the reported coverage was 13% for the over-65 population [6]

In Italy, administration strategies vary significantly across regions. This heterogeneity may produce low compliance in vaccination among the elderly, as the channel of information and of delivery may play a key role in whether the information is (a) received, (b) accepted, and (c) vaccine administered. One major goal of the present study is therefore to explore the pneumococcal vaccination coverage in the elderly population in relation to respondents’ regional area and to information sources.

Health literacy in general “encompasses people’s knowledge, motivation and competence to access, understand, appraise and apply health information in order to make judgments and take decisions in everyday life concerning health care, disease prevention, and health promotion, to maintain or improve quality of life” [7]. Since low vaccine literacy is associated with difficulties in decision-making about vaccination [8,9], it is important to verify whether patients are informed about the pneumococcal vaccine, and the extent to which the information they have is accurate. A critical aspect of vaccination literacy pertains to the information sources. The current literature has invested some effort in identifying the primary sources of information for the general population of several vaccines, including the recent COVID-19 vaccines [10] However, little is still known about the specific sources that inform the elderly about pneumococcal vaccines, a crucial piece of information to design targeted interventions aimed at increasing vaccine coverage by decreasing vaccine hesitancy.

Vaccine hesitancy can be defined as an intermediate position in a continuum between the two extreme poles of those who fully trust and embrace vaccination and those that absolutely refuse it [11]. Vaccine hesitancy entails behavioural delays, explicit concerns about safety, and ambivalent attitudes. Among the motivations behind vaccine hesitancy, MacDonand [12] identifies factors related to the assessment of the vaccine, to risk perception (and specifically the optimism regarding the probability of getting the disease), and regarding the accessibility to vaccination, with seeking information playing a major role. Vaccine hesitancy can be influenced by the lack of correct information for a number of reasons. As Rowlands [13] reported, information about vaccination is complex: for example, there are several different vaccines addressing different diseases, and patients take into account different sources of risk, such as risk of infections, the severity of potential infections, the risk of adverse reactions to the vaccination and the severity of such reactions. When patients are not clearly informed, their uncertainty can lead to inaction. This is specifically relevant for vaccinations that are characterized by high administration complexity: For example, because they involve multiple doses, which leave space for communication inconsistencies and consequent confusion. In the absence of a uniform way of administration and of communication, it is likely that the framing of these vaccinations is not consistent. It is reasonable to assume that the PCV-13 vaccine is sometimes proposed as a vaccine against Pneumococcus, whereas the PPSV-23 is sometimes framed as a “recall” or “booster dose” due to the fact that it is generally administered after the PCV-13. The linguistic framing of these vaccines represents an important challenge from a psychological perspective, as a booster dose may be perceived as less important than the primary dose (and therefore neglected, [14]). Indeed, patients may believe that the first vaccine is still offering protection, feeding the general illusion that they are less likely to be at risk compared to other people (optimistic bias, [15]).

This cross-sectional study explores the self-reported pneumococcal vaccine status of the elderly across different regions in Italy and explores individual and institutional promoters and barriers for vaccination against pneumococcal diseases. Specifically, the aims of the study are to test and explore:

(a) Pneumococcal vaccination coverage;
(b) Whether older adults are informed about pneumococcal vaccines (Vaccine literacy);
(c) Which are the primary information sources about pneumococcal vaccines;
(d) Which are the sources more associated with vaccination acceptance;
(e) Risk perception related to pneumococcal diseases and vaccine adverse reactions;
(f) Attitudes towards pneumococcal vaccines.

## 2. Materials and Methods

### 2.1. Participants

The survey involved 600 participants who agreed to participate in the study through a phone interview. Data were collected by using the certified panel of IPSOS, which previously provided evidence of representativeness and data quality for estimating vaccination coverages [16]. Data are representative of the Italian population by gender (311 male and 289 female respondents) and geographical region and focused on a sample of participants aged between 65 and 70, M = 67.51, SD = 1.46.

The result of a sensitivity power analysis indicated that with *n* = 600 participants, we have 80% power to detect an effect of Cohen’s d > 0.11 for matched pair *t*-tests, and effect of phi > 0.11 for chi-squared tests.

Given that gender and region did not affect respondents’ answers, these two factors will no longer be discussed. Information related to participants’ geographical area is available in Table 1.

The study was approved by the Ethics Committee of University of Padova (Protocol Number: F1370ABF896EB9F2C442E17B9ED3C689).

### 2.2. Measures

After agreeing to the informed consent, participants were first asked to provide socio-demographic information (age, gender, and region of residence). Consequently, we assessed their Vaccine Literacy, asking whether they were aware of available vaccines against pneumococcal diseases, how many doses are recommended in Italy, and whether they were informed about the PVC-13 and PPSV-23 vaccines. We then asked who provided them with their information.

Sociodemographic information: Participants were asked their age, gender, and regional residence.

Vaccine literacy: Participants were asked to indicate whether they were aware that vaccines against Pneumococcus are available and to indicate how many doses are recommended in Italy. Two additional questions asked participants whether they were informed about the PVC-13 and PPSV-23.

Information Sources: Participants were asked to indicate from whom they received information about pneumococcal vaccines.

Vaccination Status: Participants were asked to indicate whether they already received the PVC-13 and/or the PPSV-23. Then, we asked vaccinated participants who administered the vaccination and asked unvaccinated participants why they did not get the vaccine.

Attitudes toward Pneumococcal vaccines: We informed all participants about the fact that to be considered fully vaccinated in Italy, first it is required to receive the PVC-13 vaccine, and then receive the PPSV-23 after about two months. Then, we assessed participants’ attitudes towards vaccines with 8 items on a 5-points Likert scale. Specifically, participants evaluated the safety, effectiveness, and usefulness of PVC-13 and PPSV-23 vaccines. As the reliability was good (α = 0.97), we averaged items’ scores. Then, we asked the participants to indicate whether PVC-13 and PPSV-23 are the main vaccines against Pneumococcus, if PVC-13 gives sufficient protection against Pneumococcus. Finally, we asked participants’ opinions about how PPSV-23 is perceived by other people (i.e., “it is necessary to get full protection from Pneumococcus”, “it is not necessary to get full protection, as PVC-13 gives enough protection”).

Risk perception: Two items on a 5-points Likert scale (adapted from McKenna, 1993) assessed participants’ risk perception related to Pneumococcal diseases and adverse reactions to vaccines.

## 3. Results

### 3.1. Vaccination Coverage

Only a minority of respondents (*n*= 67, 11.2%) reported being vaccinated with PVC-13 and/or PPSV-23. Among those who are not vaccinated, 19 (3.2%) planned to get vaccinated soon. Demographic features (i.e., gender and age) were not characterizing this pattern (all ps > 0.05). Regional area was also not associated with differences in vaccine coverages (χ^2^(8) = 9.19, *p* = 0.33). Vaccine coverages ranged from 8.2% in the south region to 16.8% in the northeast region.

### 3.2. Vaccination Literacy

The majority of respondents (62.3%, χ^2^ = 36.88, *p* < 0.001, test value = 0.50) are aware of the existence of an anti-pneumonia vaccination. Among those respondents that are aware of the anti-pneumonia vaccination (*n* = 374), we still observe a suboptimal level of knowledge, since only 24.3% reported that the number of vaccines is 2, 43.6% reported that one vaccine is required, 6.7% thought that the number of vaccines depended on their health status, 1.6 % thought that three vaccines were involved in the conjugate vaccination and the remaining 23.8% reported that they did not know about the number of required vaccines.

### 3.3. Information Sources

**Figure 1:**
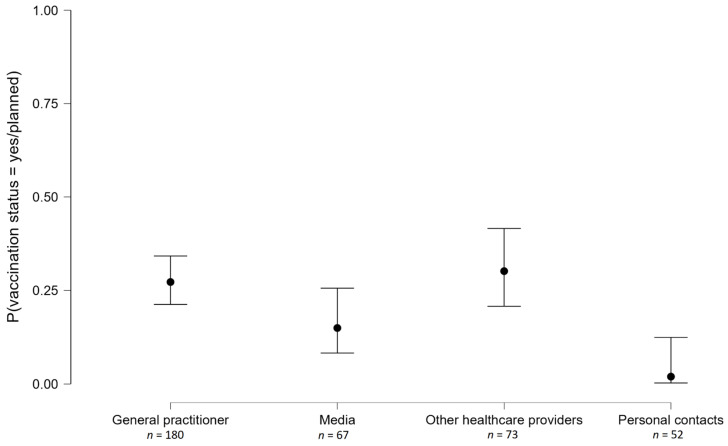
Probability of being vaccinated according to the information source.

As shown in Figure 1, 48.1% of respondents reported that their major source of information is the general practitioner (GP), the second most common source is acquaintances (13.1%), followed by the local health structure (9.4%). Only 91 respondents (over 600) reported knowing that two vaccines are required for anti-pneumonia vaccination and primarily had this information from their doctor (57.1%) and their local health structure (11%). The remaining respondents (namely 84.8% of the entire sample) had no or wrong knowledge about anti-pneumonia vaccination.

By crossing the information source (4 levels) with Vaccination Status (coded as a dichotomous variable, namely, vaccinated/planned to get vaccinated soon vs. no), we see that decision-making is differentially associated with the information source; test for the equality of proportion χ^2^ (3) = 19.82, *p* < 0.001. As represented in Figure 1, the pairwise comparisons show that respondents informed by media were less likely to be vaccinated compared to respondents informed by a GP (χ^2^ (1) = 4.06, *p* = 0.04) or by other healthcare providers (χ^2^ (1) = 4.58, *p* = 0.03), but more likely to be vaccinated compared to respondents informed by their personal contacts (χ^2^ (1) = 5.90, *p* = 0.02).

A similar pattern can be observed for the specific questions regarding PVC-13 and PPSV-23.

PVC-13: The majority of respondents (80.7%) are not informed of PCV-13, χ^2^ = 242.55, *p* < 0.0001, test value = 0.50. Participants who knew about PCV-13 were informed by their own doctor (*n* = 71, 61%) and by their local sanitary structure (12.7%). Only the minority of participants who knew about PCV-13 (*n* = 116) did actually receive it (*n* = 50, 43.1%). They received their shot from their doctor (*n* = 33), at the local sanitary structure (*n* = 12), or at the hospital (*n* = 5).

Respondents that are not vaccinated with PCV-13 reported as the main reason the fact that nobody offered them the shot (*n* = 20), they are going to take the vaccine shortly (*n* = 16) or they had already been vaccinated with PPSV-23 (*n* = 12). Only five respondents said that they do not want to get vaccinated, and only one person said that she was suggested not to. Some people [5] referred that they were vaccinated in the past. One person reported that his doctor said he did not need it.

PPSV-23: The majority of respondents (85.0%) are not informed about PPSV-23, χ^2^ = 324.53, *p* < 0.0001, test value = 0.50. The majority of participants who knew about PPSV-23 (*n* = 90) did actually receive it (*n* = 56, 62.22), χ^2^ = 5.43, *p* = 0.02. They received their shot from their doctor (*n* = 32), at the local sanitary structure (*n* = 18), or at the hospital (*n* = 6). Respondents that are not vaccinated with PPSV-23 reported as the main reason the fact that nobody offered them the shot (*n* = 8), they are going to take the vaccine shortly (*n* = 12) or they had already been vaccinated for PCV-13 (*n* = 1). Only three respondents said that they do not want to get vaccinated, one person has not yet decided if she wants to get the vaccine, and one person said that she has no pathologies.

### 3.4. Risk Perception

The estimated probabilities of suffering pneumococcus (M = 2.41; SD = 0.97, one-sample *t* = −14.82, *p* < 0.0001, comparison score 3) and to experience negative reactions to the vaccine (M = 2.41; SD = 1.02, one sample *t* = −14.39, *p* < 0.0001, comparison score 3) show that respondents underestimated their probability of both negative events in comparison to other people, suggesting the presence of an optimistic bias.

### 3.5. Attitudes towards Vaccines

Respondents evaluated PCV-13 and PPSV-23 according to four dimensions (effectiveness, safety, utility, and whether it is the main vaccine). Responses were analysed with a full factorial mixed linear model, with respondents’ ID as a random factor, and type of vaccine and dimensions as factors.

PPSV-23 (M = 3.97, SD = 0.98) received lower scores than PCV-13 (M = 3.96; SD = 0.97), F(1, 599) = 25.90, *p* < 0.0001. A main effect of evaluative dimension, F(3, 1797) = 10.40, *p* < 0.0001, was further characterized by the type of vaccine, F(3, 1797) = 4.52, *p* = 0.0036. The interaction was inspected through pairwise post hoc comparisons with Tuckey correction. Focusing on the contrast between the two vaccines, PCV-13 was perceived as more useful (MPCV-13 = 4.04; SD PCV-13 = 0.99; MPPSV-23 = 3.95; SD PPSV-23 = 1, *p* < 0.001), as the main vaccination (MPCV-13 = 3.97 SD PCV-13 = 0.95; MPPSV-23 = 3.88; SD PPSV-23 = 0.97; *p* < 0.0001) and as more effective (MPCV-13 = 3.95; SD PCV-13 = 0.94; MPPSV-23 = 3.90; SD PPSV-23 = 0.97; *p* = 0.055). The vaccines were similarly evaluated for safety (MPCV-13 = 3.90; SD PCV-13 = 0.99; MPPSV-23 = 3.86; SD PPSV-23 = 0.99; *p* = 0.60).

When asked to indicate their opinion about vaccine protection after PCV-13, most respondents (62.3%) agreed with the sentence that there is a good protection, but PPSV-23 is needed to obtain maximum protection. In total, 23% agreed with the assertion that there is a good protection and PPSV-23 is not necessary. A total of 14.7% agreed that PCV-13 is definitely not enough.

**Figure 2:**
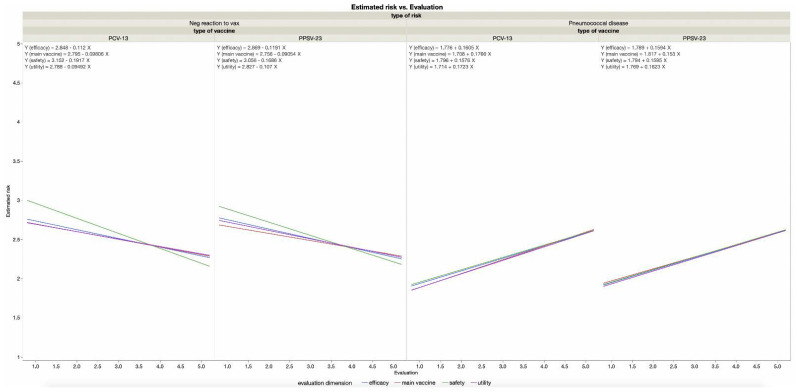
Participants’ estimated risk of getting sick and of negative reactions to vaccination, in relation to evaluations of PCV-13 and PPSV-23.3.1.

When asked to indicate what people think, the majority of respondents (69.5%) selected the option according to which PPSV-23 is needed to obtain the highest protection. One-third of respondents (30.5%) selected the option that the PPSV-23 is not needed since PCV-13 is enough. The assumption that PPSV-23 is unnecessary is more attributed to others than endorsed by the self. We further included in the model participants’ estimated probability for the self (compared to others) to experience pneumococcus and to experience negative reactions to the vaccine. As we can see in Figure 2, higher risk perception for being infected with pneumococcus and lower perception for experiencing vaccine reactions are associated with perceiving vaccines as safer, useful, effective, and being the main vaccine to prevent pneumococcus infection (all *p*s ≤ 0.03, regression coefficients are reported in Figure 2).

## 4. Discussion

Since self-reported vaccination status can be considered a reliable proxy of actual coverage [17] and we used a representative sample of the Italian 65-to-70 years old population, this study provides the first estimate of the pneumococcal vaccination coverage across Italian regions. Specifically, we found that vaccine coverage for pneumococcal vaccination is 11.2%. Moreover, we did not find statistically significant differences based on participants’ region and gender.

In addition, the data show that vaccine literacy is extremely low (and this is independent of geographical region and gender) and that the most accessible and most effective source of information is the general practitioner (GP) and other health providers. Moreover, from the qualitative analyses of the reported reasons for not being vaccinated, one prevalent motivation was that nobody actually offered the vaccine. Together, the results suggest that vaccination coverage could be enhanced if the system relied less on active demand for vaccination (i.e., adherence by an informed public) and more on passive acceptance of vaccinations, namely compliance to explicit recommendations [18]. We therefore suggest implementing interventions to mobilize GPs in active campaigns with their patients, as GPs are the prevalent source of information for vaccinated respondents [19,20,21]. This suggestion is in line with the data of an intervention study conducted in Singapore [22], where GPs were involved in a cluster-randomized crossover trial involving 9000 patients older than 65 in two time periods, namely the intervention and the control period. During the intervention, the practitioners provided their patients with information (e.g., flyers, posters) about pneumococcal disease and vaccines, which led to an increase (compared to a control group) in pneumococcal vaccine uptake (5.7% vs. 3.7%, *p* = 0.001). Importantly, the proposed interventions may have cascade effects, since informed patients may become, in turn, sources of information for others, spreading vaccine literacy and stimulating immunization normative [23,24,25]. Indeed, personal contacts are generally not involved in our respondents’ knowledge of pneumococcal vaccination, and they are more likely reported as a source by non-vaccinated respondents. Future studies may verify whether this trend can be reversed in a context where significant others are more knowledgeable. If this were the case, spreading information in the social context may trigger a virtuous circle that protects against the many fake news and conspiracy theories that target vaccines [26,27,28].

A further issue concerns the linguistic framing of the vaccines. Interestingly, participants have different attitudes towards the two vaccines: compared to PPSV-23, the PCV-13 was evaluated as more effective, useful, and as the main vaccine. While it is possible to hypothesize that PCV-13 received more positive evaluations due to the lower number of antigens contained compared to the PPSV-23, this interpretation seems unlikely as the attitude about safety was similar for both vaccines. We rather interpret these differences based on the linguistic and pragmatic framing of the two. Indeed, PCV-13 is generally linguistically and procedurally presented as the temporally first vaccine. Literature shows that what is first presented is also perceived as preeminent [29], consequently, it is possible that PPSV-23 can be seen as an unnecessary second dose or as a recall of the first vaccine. Coherently, 25% of participants reported that PCV-13 already provides the required protection against pneumococcus. The frame of communication may be also relevant for other vaccinations that require more than one dose (e.g., many vaccinations currently implemented against COVID-19).

Moreover, our results corroborated the extant knowledge about the vaccine hesitancy underpinnings, in particular about the key role of risk perception [30]: The more the infection was perceived as likely, the more participants positively evaluated both vaccines. Complementarily, the risk perception of vaccine-related adverse reactions was associated with negative attitudes toward both vaccines. Therefore, information campaigns should carefully consider both sources of risk as they are important, yet divergent predictors, of vaccination hesitancy.

Our work had important strengths. First, it is the first time that data about Pneumococcus vaccination National coverage are publicly released in the Italian context. Moreover, this type of data is very scarce, especially if we take into account the international arena. Importantly, the reported data involved a representative sample of the population under investigation and specifically focused on a targeted age group. Finally, we were able to evaluate the socio-cognitive underpinnings of attitudes towards this type of vaccination, offering important insights for policy makers and communicators.

However, the study also had limitations that should be overcome with future empirical efforts. In particular, we have no information about the educational level of our sample or about its political leaning, which might be relevant pieces of information [31]. Additionally, the comparison between attitudes and knowledge toward different type of vaccinations would offer additional insights about the specificity of vaccine hesitancy and lack of literacy for Pneumococcus vaccination compared to other relevant vaccinations (e.g., Herpes Zooster or Influenza).

Finally, while focusing on a specific age range (65 to 70 years old) improved the reliability of our estimates on that specific population, it was noted that our results are not informative for older individuals. It is possible that older individuals are more likely to be in contact with healthcare practitioners and therefore more informed about vaccinations.

However, both CDC and the Italian Ministry of Health recommend anti-pneumococcus vaccinations from 65 years old, therefore estimates related to this specific age group are particularly important to detect timely compliance with public health guidelines.

## 5. Conclusions

The present study pictured a scenario where the Italian elder population not only is not sufficiently covered by pneumococcal vaccinations, but it is not even informed about vaccination opportunities and recommendations, calling for educational campaigns. The results suggest that the main source of information should be GPs and other healthcare providers. In line with the Health Beliefs Model [32], the content of the informational messages should entail both appealing to the risk of contracting pneumonia (appealing to fear) and reassuring patients about vaccination safety (proposed solution). While this study focused on the Italian elder population and specific vaccinations against pneumococcus, it is worth pointing out that it is plausible that the processes highlighted in this report may be relevant for other countries and other vaccines targeting different infectious diseases. Indeed, previous studies conducted in different countries highlighted suboptimal levels of coverages for both anti-pneumococcus and anti-influenza vaccinations [4,5]. Thus, future studies are required in order to generalize our results in different contexts and for different vaccines.

## Figures and Tables

**Table 1:** Participants’ geographical Area.

Geographical Area	Frequency	Percent
Centre	119	19.833
Isles	63	10.500
Northeast	119	19.833
Northwest	164	27.333
South	135	22.500

## Data Availability

The data presented in this study are openly available in OSF at osf.io/pdmx6.

## References

[1] Gusmano M.K., Michel J.P. (2009). Life course vaccination and healthy aging. Aging Clin. Exp. Res..

[2] Rechel B., Priaulx J., Richardson E., McKee M. (2019). The organization and delivery of vaccination services in the European Union. Eur. J. Public Health.

[3] Gilchrist S.A.N., Nanni A., Levine O. (2012). Benefits and effectiveness of administering pneumococcal polysaccharide vaccine with seasonal influenza vaccine: An approach for policymakers. Am. J. Public Health.

[4] Kohlhammer Y., Schnoor M., Schwartz M., Raspe H., Schäfer T. (2007). Determinants of influenza and pneumococcal vaccination in elderly people: A systematic review. Public Health.

[5] Vila-Córcoles A., Ochoa-Gondar O., de Diego C., Satué E., Vila-Rovira A., Aragón M. (2019). Pneumococcal vaccination coverages by age, sex and specific underlying risk conditions among middle-aged and older adults in Catalonia, Spain, 2017. Eurosurveillance.

[6] Noale M., Trevisan C., Maggi S., Incalzi R.A., Pedone C., Di Bari M., Adorni F., Jesuthasan N., Sojic A., Galli M. (2020). The association between influenza and pneumococcal vaccinations and sars-cov-2 infection: Data from the epicovid19 web-based survey. Vaccines.

[7] Michel J.P., Goldberg J. (2021). Education, Healthy Ageing and Vaccine Literacy. J. Nutr. Health Aging.

[8] Lorini C., Santomauro F., Donzellini M., Capecchi L., Bechini A., Boccalini S., Bonanni P., Bonaccorsi G. (2018). Health literacy and vaccination: A systematic review. Hum. Vaccines Immunother..

[9] Zhang F., Or P.P.L., Chung J.W.Y. (2020). The effects of health literacy in influenza vaccination competencies among community-dwelling older adults in Hong Kong. BMC Geriatr..

[10] Murphy J., Vallières F., Bentall R.P., Shevlin M., McBride O., Hartman T.K., McKay R., Bennett K., Mason L., Gibson-Miller J. (2021). Psychological characteristics associated with COVID-19 vaccine hesitancy and resistance in Ireland and the United Kingdom. Nat. Commun..

[11] Dubé E., Laberge C., Guay M., Bramadat P., Roy R., Bettinger J. (2013). Vaccine hesitancy: An overview. Hum. Vaccines Immunother..

[12] MacDonald N.E., Eskola J., Liang X., Chaudhuri M., Dube E., Gellin B., Goldstein S., Larson H., Manzo M.L., Reingold A. (2015). Vaccine hesitancy: Definition, scope and determinants. Vaccine.

[13] Rowlands G. (2014). Health literacy: Ways to maximise the impact and effectiveness of vaccination information. Hum. Vaccines Immunother..

[14] Riccò M., Cattani S., Veronesi L., Colucci M.E. (2016). Knowledge, attitudes, beliefs and practices of construction workers towards tetanus vaccine in northern Italy. Ind. Health.

[15] McKenna F.P. (1993). It won’t happen to me: Unrealistic optimism or illusion of control?. Br. J. Psychol..

[16] Bradley V.C., Kuriwaki S., Isakov M., Sejdinovic D., Meng X.L., Flaxman S. (2021). Unrepresentative big surveys significantly overestimated US vaccine uptake. Nature.

[17] Mac Donald R., Baken L., Nelson A., Nichol K.L. (1999). Validation of self-report of influenza and pneumococcal vaccination status in elderly outpatients. Am. J. Prev. Med..

[18] Nichter M. (1995). Vaccinations in the third world: A consideration of community demand. Soc. Sci. Med..

[19] Kane M.A. (1998). Commentary: Public perception and the safety of immunization. Vaccine.

[20] Schmitt H.J., Booy R., Aston R., Van Damme P., Schumacher R.F., Campins M., Rodrigo C., Heikkinen T., Weil-Olivier C., Finn A. (2007). How to optimise the coverage rate of infant and adult immunisations in Europe. BMC Med..

[21] Ridda I., Motbey C., Lam L., Lindley I.R., McIntyre P.B., MacIntyre C.R. (2008). Factors associated with pneumococcal immunisation among hospitalised elderly persons: A survey of patient’s perception, attitude, and knowledge. Vaccine.

[22] Ho H.J., Tan Y.R., Cook A.R., Koh G., Tham T.Y., Anwar E., Chiang G.S.H., Lwin M.O., Chen M.I. (2019). Increasing influenza and pneumococcal vaccination uptake in seniors using point-of-care informational interventions in primary care in Singapore: A pragmatic, cluster-randomized crossover trial. Am. J. Public Health.

[23] Kempe A., Patel M.M., Daley M.F., Crane L.A., Beaty B., Stokley S., Barrow J., Babbel C., Dickinson L.M., Tempte J.L. (2009). Adoption of rotavirus vaccination by pediatricians and family medicine physicians in the United States. Pediatrics.

[24] Gilca V., Boulianne N., Dubé E., Sauvageau C., Ouakki M. (2009). Attitudes of nurses toward current and proposed vaccines for public programs: A questionnaire survey. Int. J. Nurs. Stud..

[25] Harmsen I.A., Lambooij M.S., Ruiter R.A.C., Mollema L., Veldwijk J., van Weert Y.J.W.M., Kok G., Paulussen T.G.W., de Wit G.A., de Melker H.E. (2012). Psychosocial determinants of parents’ intention to vaccinate their newborn child against hepatitis B. Vaccine.

[26] Wolfe R.M., Sharp L.K., Lipsky M.S. (2002). Content and design attributes of antivaccination Web sites. J. Am. Med. Assoc..

[27] Diethelm P., McKee M. (2009). Denialism: What is it and how should scientists respond?. Eur. J. Public Health.

[28] Kata A. (2012). Anti-vaccine activists, Web 2.0, and the postmodern paradigm—An overview of tactics and tropes used online by the anti-vaccination movement. Vaccine.

[29] Bettinsoli M.L., Maass A., Kashima Y., Suitner C. (2015). Word-order and causal inference: The temporal attribution bias. J. Exp. Soc. Psychol..

[30] Caserotti M., Girardi P., Rubaltelli E., Tasso A., Lotto L., Gavaruzzi T. (2021). Associations of COVID-19 risk perception with vaccine hesitancy over time for Italian residents. Soc. Sci. Med..

[31] Dubé È., Ward J.K., Verger P., Macdonald N.E. (2020). Vaccine Hesitancy, Acceptance, and Anti-Vaccination: Trends and Future Prospects for Public Health. Annu. Rev. Public Health.

[32] Rosenstock I.M. (1977). The Health Belief Model and Preventive Health Behavior. Health Educ. Monogr..

